# Mechanical interactions in bacterial colonies and the surfing probability of beneficial mutations

**DOI:** 10.1098/rsif.2017.0073

**Published:** 2017-06-07

**Authors:** Fred D. Farrell, Matti Gralka, Oskar Hallatschek, Bartlomiej Waclaw

**Affiliations:** 1 Life Sciences, University of Warwick, Coventry CV4 7AL, UK; 2 Department of Physics, University of California, Berkeley, CA 94720, USA; 3 Department of Integrative Biology, University of California, Berkeley, CA 94720, USA; 4 School of Physics and Astronomy, University of Edinburgh, JCMB, Peter Guthrie Tait Road, Edinburgh EH9 3FD, UK; 5 Centre for Synthetic and Systems Biology, University of Edinburgh, CH Waddington Building, Max Born Crescent, Edinburgh EH9 3BF, UK

**Keywords:** biological evolution, bacterial colony, interactions, surfing probability, roughness

## Abstract

Bacterial conglomerates such as biofilms and microcolonies are ubiquitous in nature and play an important role in industry and medicine. In contrast to well-mixed cultures routinely used in microbial research, bacteria in a microcolony interact mechanically with one another and with the substrate to which they are attached. Here, we use a computer model of a microbial colony of rod-shaped cells to investigate how physical interactions between cells determine their motion in the colony and how this affects biological evolution. We show that the probability that a faster-growing mutant ‘surfs’ at the colony's frontier and creates a macroscopic sector depends on physical properties of cells (shape, elasticity and friction). Although all these factors contribute to the surfing probability in seemingly different ways, their effects can be summarized by two summary statistics that characterize the front roughness and cell alignment. Our predictions are confirmed by experiments in which we measure the surfing probability for colonies of different front roughness. Our results show that physical interactions between bacterial cells play an important role in biological evolution of new traits, and suggest that these interactions may be relevant to processes such as *de novo* evolution of antibiotic resistance.

## 1. Introduction

Bacteria are the most numerous organisms on Earth capable of autonomous reproduction. They have colonized virtually all ecological niches and are able to survive harsh conditions intolerable for other organisms such as high salinity, low pH, extreme temperatures, or the presence of toxic elements and compounds [1]. Many bacteria are important animal or human pathogens, but some bacteria find applications in industry as waste degraders [2] or to produce fuels and chemicals [3]. In these roles, biological evolution of microbes is usually an undesired side effect, because it can disrupt industrial processes or lead to the emergence of new pathogenic [4] or antibiotic-resistant strains [5].

Experimental research on bacterial evolution has been traditionally carried out in well-stirred cultures [6,7]. However, in their natural environment, bacteria often form aggregates such as microcolonies and biofilms. Such aggregates can be found on food [8], teeth (plaque), on catheters or surgical implants [9], inside water distribution pipes [10] or in the lungs of people affected by cystic fibrosis [11]. Bacteria in these aggregates adhere to one another and the surface on which they live, form layers of reduced permeability to detergents and drugs, and stochastically switch to a different phenotype that is more resistant to treatment [12–14]; this causes biofilms to be notoriously difficult to remove.

An important aspect of bacteria living in dense conglomerates is that they do not only interact via chemical signalling such as quorum sensing [15] but also through mechanical forces such as when they push away or drag other bacteria when sliding past them. Computer simulations [16–19] and experiments [20–24] have indicated that such mechanical interactions play an important role in determining how microbial colonies grow and what shape they assume. However, the impact of these interactions on biological evolution only recently came into focus [25].

A particularly interesting scenario relevant to microbial evolution in microcolonies and biofilms is that of a range expansion [26] in which a population of microbes invades a new territory. If a new genetic variant arises near the invasion front, it either ‘surfs’ on the front and spreads into the new territory, or (if unlucky) it lags behind the front and forms only a small ‘bubble’ in the bulk of the population [27]. This stochastic process, called ‘gene surfing’, has been extensively studied [25,28–34], but these works have not addressed the role of mechanical interactions between cells. Many of the existing models do not consider individual cells [28], assume Eden-like growth [32], or are only appropriate for diluted populations of motile cells described by reaction–diffusion equations similar to the Fisher–Kolmogorov equation [35]. On the other hand, agent-based models of biofilm growth, which have been applied to study biological evolution in growing biofilms [36–38], use very simple rules to mimic cell–cell repulsion which neglect important physical aspects of cell–cell and cell–substrate interactions such as friction.

In this work, we use a computer model of a growing microbial colony to study how gene surfing is affected by the mechanical properties of cells and their environment. In our model, non-motile bacteria grow attached to a two-dimensional permeable surface which delivers nutrients to the colony. This corresponds to a common experimental scenario in which bacteria grow on the surface of agarose gel infused with nutrients. We have previously demonstrated [17] that this model predicts a non-equilibrium phase transition between a regular (circular) and irregular (branched) shape of a radially expanding colony of microbes, and that it can be used to study biological evolution in microbial colonies [25]. Here, we use this model to show that the surfing probability of a beneficial mutation is determined by the roughness and the cellular ordering at the expanding front of the colony. We also investigate how mechanical properties of cells, such as elasticity, friction and cell shape, affect these two quantities. We corroborate some of our results in experiments with microbial colonies that display varying degrees of roughness of the growing front and show that it influences the surfing probability as expected.

## 2. Computer model

We use a computer model similar to that from [17,23,25], with some modifications. Here, we discuss only the generic algorithm; more details will be given in subsequent sections where we shall talk about the role of each of the mechanical factors.

**Figure 1.**
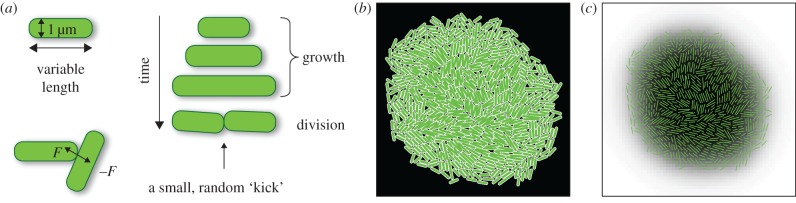
(*a*) Illustration of the computer algorithm. Bacteria are modelled as rods of varying length and constant diameter. When a growing rod exceeds a critical length, it splits into two smaller rods. (*b*) A small simulated colony. (*c*) The same colony with nutrient concentration shown as different shades of grey (white, maximal concentration; black, minimal); the cells are represented as thin green lines. (Online version in colour.)

We assume that bacteria form a monolayer as if the colony was two dimensional and bacteria always remained attached to the substrate. This is a good approximation to what occurs at the edge of the colony and, as we shall see, is entirely justifiable because the edge is the part of the colony most relevant for biological evolution of new traits. We model cells as spherocylinders of variable length and constant diameter *d* = 2*r*_0_ = 1 μm (figure 1*a*). Cells repel each other with normal force determined by the Hertzian contact theory: *F* = (4/3)*Er*^1/2^_0_*h*^3/2^, where *h* is the overlap distance between the walls of the interacting cells and *E* plays the role of the elastic modulus of the cell. The dynamics is overdamped, i.e. the linear/angular velocity is proportional to the total force/total torque acting on the cell:2.1
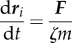
and2.2
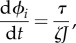
where ***r***_*i*_ is the position of the centre of mass of cell *i*, *ϕ*_*i*_ is the angle it makes with the *x*-axis, ***F*** and *τ* are the total force and torque acting on the cell, *m* and *J* are its mass and the momentum of inertia (perpendicular to the plane of growth), respectively, and *ζ* is the damping (friction) coefficient. We initially assume that friction is isotropic and explore anisotropic friction later in §4.3. Note that the mass *m* and the momentum of inertia *J* are the proxy for cell size. These quantities are not constant because cells change their size over time, and hence *m*, *J* cannot be absorbed into the friction coefficient.

Bacteria grow by consuming nutrients that diffuse in the substrate. The limiting nutrient concentration dynamics is modelled by the diffusion equation with sinks corresponding to the bacteria consuming the nutrient:2.3
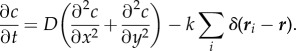
Here, ***r*** = (*x*, *y*), *c* = *c*(***r***, *t*) is the nutrient concentration at position ***r*** and time *t*, *D* is the diffusion coefficient of the nutrient and *k* is the nutrient uptake rate. The initial concentration *c*(***r***, 0) = *c*_0_.

A cell elongates at a constant rate *v*_*l*_ as long as the local nutrient concentration is larger than a certain fraction (more than 1%) of the initial concentration. When a growing cell reaches a predetermined length, it divides into two daughter cells whose lengths are half the length of the mother cell. The critical inter-cap distance *l*_cap-cap_ at which this occurs is a random variable from a Gaussian distribution with mean ℓ_*c*_ and standard deviation ±0.15 ℓ_*c*_. Varying ℓ_*c*_ allows us to extrapolate between quasi-spherical cells (e.g. yeasts *S. cerevisiae* or the bacterium *S. aureus*) and rod-shaped cells (e.g. *Escherichia coli* or *P. aeruginosa*), whereas the randomness of *l*_cap-cap_ accounts for the loss of synchrony in replication that occurs after a few generations (the coefficient of variation of the time to division approximately 0.1–0.2 [39–41]). The two daughter cells have the same orientation as the parent cell, plus a small random perturbation to prevent the cells from growing in a straight line.

**Figure 2.**
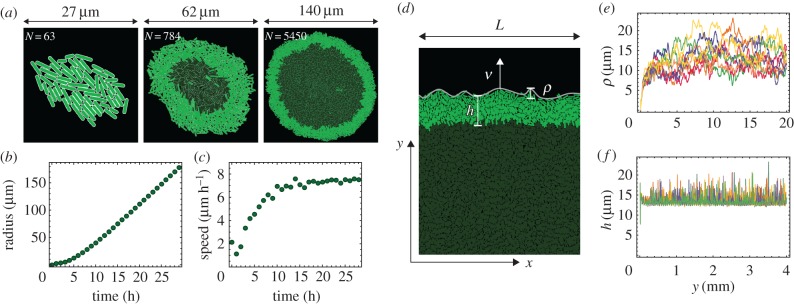
(*a*) Snapshots of a radially growing simulated colony taken at different times (sizes), for *k* = 2. Growing bacteria are bright green, quiescent (non-growing) bacteria are dark green. (*b*) The radius of the colony increases approximately linearly in time. (*c*) The expansion speed tends to a constant value for long times. (*d*) Example configuration of cells from a simulation in a tube of width *L* = 80 μm. The colony expands vertically; *h* is the thickness of the growing layer (equation (4.1)) and *ρ* is the roughness of the front (equation (4.2)). (*e*,*f*) Roughness *ρ* and thickness *h* as functions of the position *y* of the front, for *L* = 1280 μm and *k* = 2.5, and for 10 independent simulation runs (different colours). (Online version in colour.)

We use two geometries in our simulations: a radially expanding colony that starts from a single bacterium (figure 2*a*), and a colony growing in a narrow (width *L*) but infinitely long vertical tube with periodic boundary conditions in the direction lateral to the expanding front (figure 2*d*). While the radial expansion case represents a typical experimental scenario, only relatively small colonies (10^6^ cells as opposed to more than 10^8^ cells in a real colony [25]) can be simulated in this way due to the high computational cost. The second method (growth in a tube) enables us to simulate growth for longer periods of time at the expense of confining the colony to a narrow strip and removing the curvature of the growing front. This has, however, little effect on the surfing probability of faster-growing mutants if the width *L* of the tube is sufficiently large [42].

Figure 1*b* shows a snapshot of a small colony; the concentration of the limiting nutrient is also shown. Table 1 shows default values of all parameters used in the simulation. Many of these parameters have been taken from the literature data for the bacterium *E. coli* [25], but some parameters such as the damping coefficient must be estimated indirectly [17]. We note that the assumed value of the diffusion constant *D* is unrealistically small; the actual value for small nutrient molecules such as sugars and amino acids would be approximately 10^6^ μm^2^ h^–1^, i.e. four orders of magnitude larger. Our choice of *D* is a compromise between realism and computational cost; we have also shown in [17] that the precise value of the diffusion coefficient is irrelevant in the region of parameter space which we are interested in here. We also note that in reality cessation of growth in the centre of the colony and the emergence of the growing layer may be due to the accumulation of waste chemicals, pH change, etc., rather than nutrient exhaustion. Here, we focus on the mechanical aspects of growing colonies and do not aim at reproducing the exact biochemistry of microbial cells, as long as the simulation leads to the formation of a well-defined growth layer (as observed experimentally).

**Table 1.** Default values of the parameters of the model. This gives ≈30 min doubling time and the average length of bacterium ≈3 μm. If not indicated otherwise, all results presented have been obtained using these parameters.

name	value	units
nutrient diffusion constant *D*	50	μm^2^ h^−1^
nutrient concentration *c*_0_	1	a.u.
nutrient uptake rate *k*	1–3	a.u. h^−1^
Young's modulus *E*	100	kPa
elongation length *v*_*l*_	4	μm h^−1^
cell diameter	1	μm
average max. inter-cap distance *l*_*c*_	4	μm
damping coefficient *ζ*	500	Pa h

## 3. Experiments

Experiments were performed as described in our previous work [25]. Here we provide a brief description of these methods.

### 3.1. Strains and growth conditions

For the mixture experiments measuring surfing probability, we used pairs of microbial strains that differed in fluorescence colour and a selectable marker. The selective difference between the strains was adjusted as in [25] using low doses of antibiotics. The background strains and antibiotics used were *E. coli* DH5*α* with tetracycline, *E. coli* MG1655 with chloramphenicol and *S. cerevisiae* W303 with cycloheximide. Selective differences were measured using the colliding colony assay [33]. *E. coli* strains were grown on LB agar (2%) medium (10 g l^−1^ tryptone, 5 g l^−1^ yeast extract, 10 g l^−1^ NaCl) at either 37°C or 21°C. *S. cerevisiae* experiments were performed on either YPD (20 g l^−1^ peptone, 10 g l^−1^ yeast extract, 20 g l^−1^ glucose) or CSM (0.79 g l^−1^ CSM (Sunrise media Inc.), 20 g l^−1^ glucose) at 30°C. Agar 20 g l^−1^ was added to media before autoclaving. Antibiotics were added after autoclaving and cooling of the media to below 60°C.

### 3.2. Measuring surfing probability

For each pair of mutant and wild-type, a mixed starting population was prepared that contained a low initial frequency *P*_*i*_ of mutants having a selective advantage *s*. Colony growth was initiated by placing 2 μl of the mixtures onto plates and incubated until the desired final population size was reached. The initial droplet radius was measured to compute the number of cells at the droplet perimeter. The resulting colonies were imaged with a Zeiss AxioZoom v16. The number of sectors was determined by eye. The surfing probability was calculated using equation (5.1).

### 3.3. Time-lapse movies

For single cell-scale time-lapse movies, we used a Zeiss LSM700 confocal microscope with a stage-top incubator to image the first few layers of most advanced cells in growing *S. cerevisiae* and *E. coli* colonies between a coverslip and an agar pad for about 4 h, taking an image every minute.

### 3.4. Measuring roughness

Images of at least 10 equal-sized colonies per condition were segmented and the boundary detected. The squared radial distance *δr*^2^ between boundary curve and the best-fit circle to the colony was measured as a function of the angle and averaged over all possible windows of length *l*. The resulting mean *δr*^2^ was averaged over different colonies.

Images of moving fronts at the single-cell level from the time-lapse movies were first segmented using a local adaptative threshold algorithm to identify cells. The front was found by the outlines of cells directly at the front. For all possible windows of length *l*, a line was fitted to the front line and the mean squared distance from the best-fit line was measured, as in [28]. The resulting mean squared distance was averaged over all windows of length *l* and all frames.

## 4. Simulation results

### 4.1. Growth and statistical properties of the simulated colony

We now discuss the properties of our simulated colonies. When the colony is small, all bacteria grow and replicate. As the colony expands, the nutrient becomes depleted in the centre of the colony because diffusion of the nutrient cannot compensate its uptake by growing cells. This causes cessation of growth in the centre. When this happens, growth becomes restricted to a narrow layer at the edge of the colony; figure 2*a*, and the electronic supplementary material, video 1. The radius of the colony increases approximately linearly in time (figure 2*b*,*c*). The presence of a ‘growing layer’ of cells and the linear growth of the colony's radius agree with what has been observed experimentally [25,43].

Statistical properties of the growing layer can be conveniently studied using the ‘tube-like’ geometry. Figure 2*d* shows a typical configuration of cells at the colony's frontier (see also the electronic supplementary material, video 2). The growing layer can be characterized by its thickness *h* and roughness *ρ*, which we calculate as follows. We first rasterize the growing front of the colony using pixels of size 1 × 1 μm, and find the two edges of the front: the upper one (the colony edge) {*y*^+^_*i*_} and the lower one (the boundary between the growing and quiescent cells) {*y*^−^_*i*_}. We then calculate the average thickness as4.1
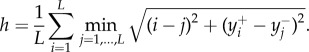
This method takes into account that the growing layer can be curved and does not have to run parallel to the *x*-axis.^1^ Similarly, we calculate the average roughness as4.2
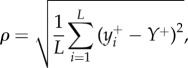
where 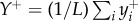. Note that all quantities (*L*, *Y*^+^, *y*^+^_*i*_, *y*^−^_*i*_) are in pixels and not μm.

After a short transient, the expansion velocity, the nutrient profile, and other properties of the growing layer stabilize and vary little with time (figure 2*e*,*f*). It is therefore convenient to choose a new reference frame co-moving with the leading edge of the colony. Since cells that lag behind the front do not replicate, we do not have to simulate these cells explicitly. This dramatically speeds up simulations and enables us to study stripes of the colony of width *L* > 1 mm and length >10 mm.

**Figure 3.**
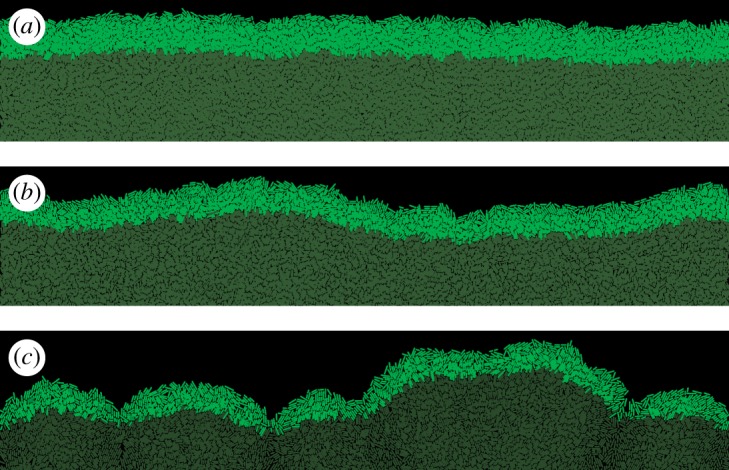
The frontier of the colony for three different nutrient uptake rates *k* = 1.8 (*a*), *k* = 2.2 (*b*) and *k* = 2.6 (*c*). The thickness of the growing layer (bright green) decreases only moderately (1.64×) from *h* = 13.5 ± 0.1 μm for *k* = 1.8 to *h* = 8.2 ± 0.1 μm for *k* = 2.6, but this has a large impact on the front roughness which changes from *ρ* = 2.1 ± 0.2 μm to *ρ* = 9.3 ± 0.4 μm, correspondingly. For *k* = 2.6, the growing layer begins to lose continuity and splits into separate branches. (Online version in colour.)

**Figure 4.**
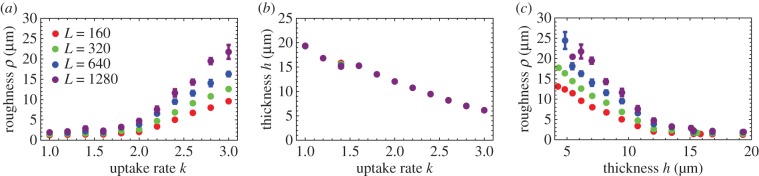
Roughness (*a*) and thickness (*b*) of the growing layer for different front lengths (tube widths) *L* = 160 (red), *L* = 320 (green), *L* = 640 (blue) and *L* = 1280 μm (purple). (*a*) Roughness *ρ* increases with both the nutrient uptake rate *k* and the length *L* of the front. (*b*) Thickness *h* decreases as *k* increases; *h* does not depend on *L*. (*c*) Roughness versus thickness; different points correspond to different *k* from panels (*a*,*b*). (Online version in colour.)

We have shown previously [17] that the thickness of the growing layer of cells is controlled by the nutrient concentration *c*_0_, nutrient uptake rate *k*, growth rate *b* and elasticity *E* of cells. This in turn affects the roughness of the leading edge of the colony. This relation is illustrated in figure 3, where we vary the uptake rate *k* while keeping the remaining parameters constant. Figure 4 shows that front thickness decreases and its roughness increases with increasing *k*; eventually, when a critical value *k*_c_ ≈ 2.5 is crossed, the growing front splits into separate branches. This transition has been investigated in detail in [17]. Although this scenario can be realized experimentally [44,45], here we focus on the ‘smooth’ regime in which colonies do not branch out and the frontier remains continuous.

### 4.2. Surfing probability of a beneficial mutation

**Figure 5.**
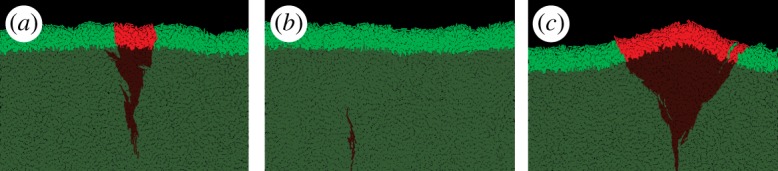
The fate of mutants. Panels (*a*) and (*b*) show different fates of a sector of fitter (*s* = 0.1) mutant cells (red) in a colony of ‘wild-type’ cells (green). The sector can either expand (*a*) or collapse and become trapped in the bulk when random fluctuations cause mutant cells to lag behind the front (*b*). Panel (*c*) shows a sector with larger (*s* = 0.5) growth advantage; significantly faster growth of mutant cells leads to a ‘bump’ at the front. In all cases, *k* = 1.8, *L* = 160 μm. (Online version in colour.)

When a mutation arises at the colony's frontier, its fate can be twofold [25,28]. If cells carrying the new mutation remain in the active layer, the mutation ‘surfs’ on the moving edge of the colony and the progeny of the mutant cell eventually forms a macroscopic ‘sector’ (figure 5). On the other hand, if cells carrying the mutation leave the active layer, the mutation becomes trapped as a ‘bubble’ in the bulk of the colony [27]. Owing to the random nature of replication and mixing at the front, surfing is a stochastic process; a mutation remains in the active layer in the limit *t* → ∞ with some probability *P*_surf_, which we shall call here the surfing probability.

Surfing is a softer version of fixation—a notion from population genetics in which a mutant takes over the population. The soft-sweep surfing probability has therefore a hard-selection-sweep counterpart, the fixation probability, which is the probability that the new mutation spreads in the population so that eventually all cells have it. Both surfing and fixation probabilities depend on the balance between selection (how well the mutant grows compared to the parent strain) and genetic drift (fluctuations in the number of organisms due to randomness in reproduction events) [46]. In [25], we showed that *P*_surf_ increased approximately linearly with selective advantage *s*—the relative difference between the growth rate of the mutant and the parent strain. Here, we study how the properties of the active layer affect *P*_surf_ for a fixed *s*.

We first run simulations in the planar-front geometry in which a random cell picked up from the growing layer of cells with probability proportional to its growth rate is replaced by a mutant cell with selective advantage *s* > 0. This can be thought of as mutations occurring with infinitely small but non-zero probability per division. The simulation finishes when either fixation (all cells in the growing layers become mutants) or extinction (no mutant cells in the growing layer) is achieved. Before inserting the mutant cell, the colony is simulated until the properties of the growing layer stabilize and both thickness and roughness reach steady-state values. The simulation is then repeated many times and the probability of surfing is estimated from the proportion of runs leading to fixation of the mutant in the growing layer. Snapshots showing different fates (extinction, surfing) of mutant sectors are shown in figure 5.

#### 4.2.1. Surfing probability depends on the position of the cell in the growing layer

**Figure 6.**
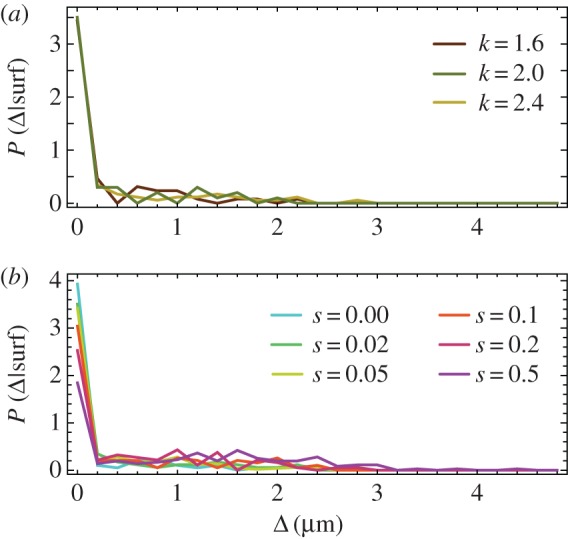
(*a*) *P*(Δ|surf) for *L* = 160 μm, selective advantage *s* = 0.02 and different *k* = 1.6, 2.0, 2.4. (*b*) *P*(Δ|surf) for *L* = 160 μm, *k* = 2.0 and different selective advantages *s* = 0, 0.02, 0.05, 0.1, 0.2, 0.5. Only mutants from the first layer of cells have a significant chance of surfing. (Online version in colour.)

In [25], we showed that the surfing probability strongly depends on how deeply in the growing layer a mutant was born. Here, we would like to emphasize this result as it will become important later. Let Δ be the distance from the edge of the colony to the place the mutant first occurred. Figure 6 shows the probability density *P*(Δ| surf) that a cell was born a distance Δ behind the colony front, *given that it went on to surf on the edge of the expanding colony*. It is evident that only cells born extremely close to the frontier have a chance to surf. Cells born farther from the frontier must get past the cells in front of them. This is unlikely to happen, even if the cell has a significant growth advantage, as the cell's growth will also tend to push forward the cells in front of it. This also justifies why we focus on two-dimensional colonies only; even though real colonies are three dimensional, all interesting dynamics occurs at the edge of the colony, which is essentially a monolayer.

Given that surfing is restricted to the first layer of cells, and the distribution *P*(Δ| surf) is approximately the same for all explored parameter sets (different *k* and *s*), for our purpose it would be a waste of computer time to simulate mutants that occurred deeply in the growing layer. To save time, and to remove the effect the front thickness has on *P*_surf_ (thicker layer = lower overall probability), we changed the way of introducing mutants. Instead of inserting mutants anywhere in the growing layer, we henceforth inserted them only at the frontier.

#### 4.2.2. Roughness of the front is more predictive of *P*_surf_ than its thickness

**Figure 7.**
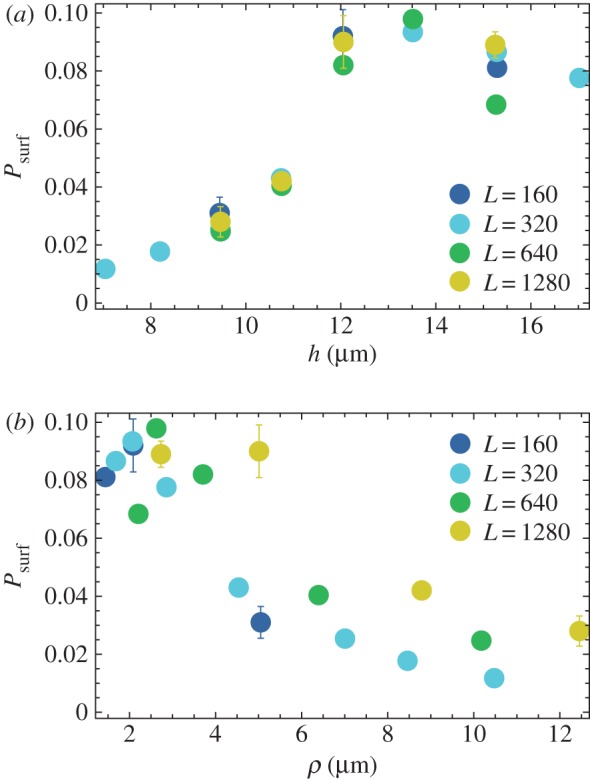
(*a*) *P*_surf_ for different thickness *h* of the growing layer, for *s* = 0.02 and *L* = 160, 320, 640, 1280 μm (different colours). (*b*) The same data as a function of front roughness *ρ*. Between 10^3^ and 10^4^ simulations were performed for each data point to estimate *P*_surf_. (Online version in colour.)

Using the new method of introducing mutants (only the first layer of cells), we run simulations for *s* = 0.02 and for different widths *L* and nutrient uptake rates *k* as in figure 4. Figure 7 shows how the surfing probability *P*_surf_ varies as a function of the thickness and the roughness of the front. *P*_surf_ increases with increasing thickness *h* and decreases with increasing roughness *ρ*. We know from figure 4 that thickness and roughness are inversely correlated, so this reciprocal behaviour is not surprising. An interesting question is whether any of the two quantities, roughness or thickness, directly affects the probability of surfing? From a statistics point of view, thickness *h* seems to be a better predictor of *P*_surf_ because data points for the same *h* but for different *L* correlate better. However, it could be that it is actually front roughness that directly (in the causal sense) affects the surfing probability and that *P*_surf_ and *h* are anti-correlated because of the relationship between *h* and *ρ*.

**Figure 8.**
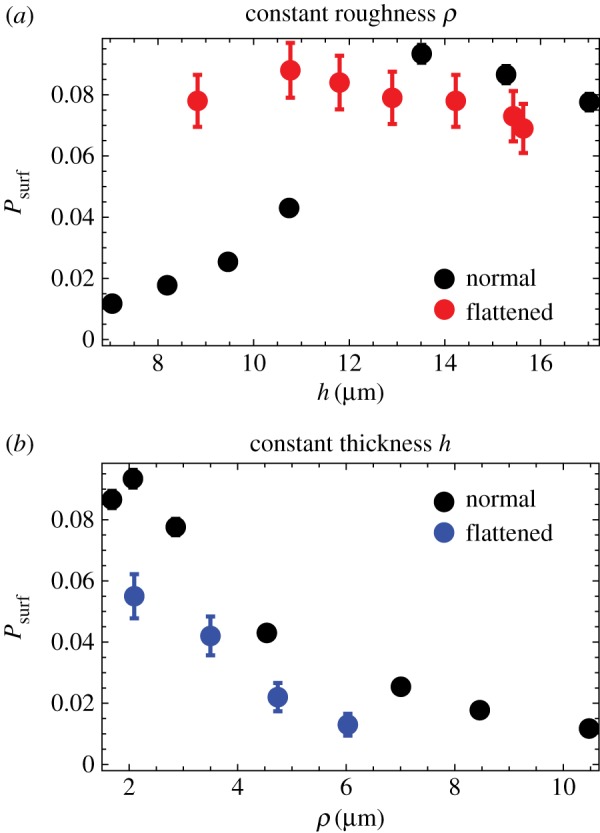
(*a*) *P*_surf_ as the function of front thickness *h* for the normal (black) and flattened front (red, *g* = 500) for *L* = 320 μm. We vary the nutrient uptake rate *k* = 1.6 … 2.8 to simulate fronts of different thickness. The flat front has roughness *ρ* between 0.84 and 1.0 for all *k*. (*b*) *P*_surf_ for the normal (black) and flattened front (blue) as the function of roughness *ρ*. The flattened front has approximately the same thickness for all data points (*h* between 10.0 and 10.3 μm). The points correspond to maximum roughness set to *ρ*_max_ = 2, 3.5, 5, and 7 μm for *k* = 2.6; the actual (measured) *ρ* differs very little from these values. (Online version in colour.)

We performed two computer experiments to address the above question. First, we simulated a colony that had a very low and constant roughness *ρ* ≈ 1 μm, independently of the front's thickness. This was achieved by introducing an external force *F*_*y*_ = −*gy* acting on the centre of mass of each cell, where *g* > 0 was a ‘flattening factor’ whose magnitude determined the strength of suppression of deviations from a flat front. *P*_surf_ plotted in figure 8*a*, as a function of *h* for two cases: ‘normal’, rough front (*g* = 0) and ‘flattened’ front (*g* > 0), demonstrates that the surfing probability does not depend on *h* in the case of flat front.

Second, we varied roughness while keeping the thickness constant. This was done by measuring front roughness in each simulation step, and switching on the external ‘flattening’ force *F*_*y*_ = −*gy* if the roughness was larger than a desired value *ρ*_max_. Figure 8*b* shows that although thickness remains the same for all data points, *P*_surf_ decreases with increasing roughness.

We can conclude from this that it is the increase in the roughness, and not decreasing thickness, that lowers the surfing probability for thinner fronts (larger nutrient intake rate *k*). However, the data points in figure 7*b*, from different simulations, do not collapse onto a single curve as it would be expected if average, large-scale front roughness was the only factor.

#### 4.2.3. Local roughness predicts *P*_surf_

**Figure 9.**
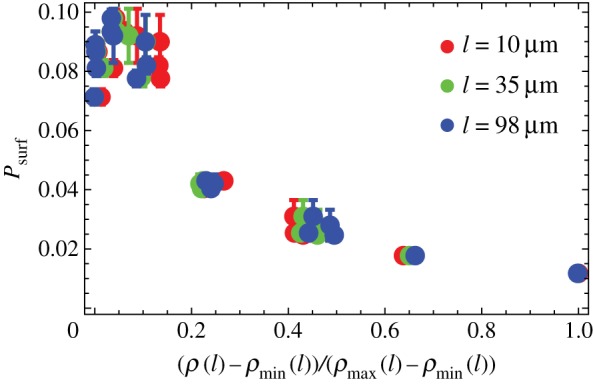
*P*_surf_ as the function of the rescaled local roughness (*ρ*(*l*) − *ρ*_min_(*l*))/(*ρ*_max_(*l*) − *ρ*_min_(*l*)) of the growing layer, for different sizes *L* = 160, 320, 640, 1280 μm (as in figure 7), *s* = 0.02, and *l* = 10 μm (red), *l* = 35 μm (green), *l* = 98 μm (blue). Data points for different *L* and *l* collapse onto a single curve. The corresponding *ρ*_max_(*l*) (the largest of the simulated *ρ*(*l*) for a given *l*) are 1.3, 3.1, 6.4 μm and *ρ*_min_(*l*) (the smallest *ρ*(*l*) for a given *l*) are 0.7, 1, 1.3 μm. (Online version in colour.)

According to the theory of ref. [30], the dynamics of a mutant sector can be described by a random process similar to Brownian motion in which the sector boundaries drift away from each other with constant velocity. The velocity depends on the growth advantage *s*, whereas the amplitude of random fluctuations in the positions of boundary walls is set by the microscopic dynamics at the front. We reasoned that these fluctuations must depend on the roughness *ρ* of the frontier, and that a mutant sector should be affected by front roughness when the sector is small compared to the magnitude of fluctuations. This means that local roughness *ρ*(*l*), determined over the length *l* of the front, should be more important than the global roughness *ρ*(*L*). We calculated the local roughness as4.3
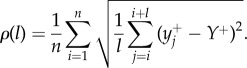
Here, *Y*^+^ is the average height of the interface and {*y*^+^_*i*_} are the vertical coordinates (interface height) of the points at the leading edge, obtained as in §4.1. Figure 9 shows that *P*_surf_ for different *L* now collapse onto a single curve, for all lengths *l* ≈ 10 ··· 100 μm over which roughness has been calculated.

#### 4.2.4. Orientation of cells affects *P*_surf_

**Figure 10.**
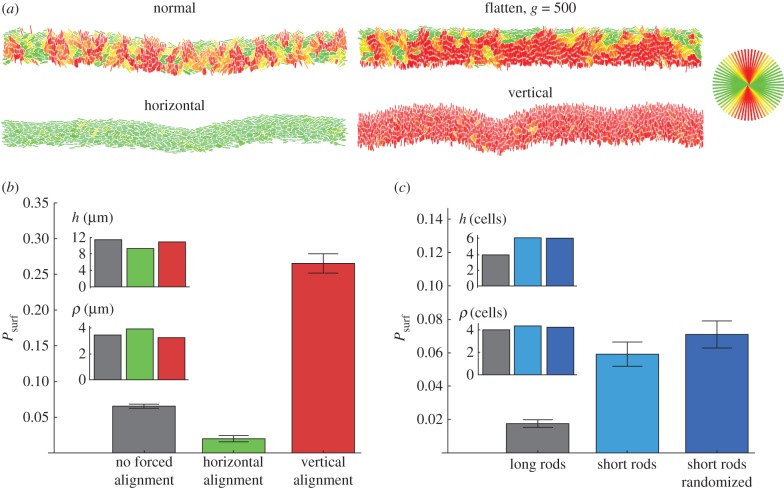
(*a*) Orientation of cells (colours as in the circle in the upper-right corner) in the growing layer for different models. (*b*,*c*) Surfing probabilities for different cellular alignments at the front, for approximately the same thickness and roughness, both of which were controlled by varying *k*. To achieve this, different *k* needed to be used in panels (*b*,*c*) and hence the two panels cannot be directly compared. In all cases *L* = 320 μm, *s* = 0.02. For horizontally and vertically forced cells, *τ*_max_ = 10 000. Short cells have a maximum length of 2 μm; upon division, they become circles of diameter 1 μm. (Online version in colour.)

So far we have focused only on the macroscopic properties of the leading edge of the colony, completely neglecting its granular nature due to the presence of individual cells. Recall that, in our model, each cell is rod-shaped, and the direction in which it grows is determined by the orientation of the rod. Figure 10*a* shows that cells at the leading edge assume orientations slightly more parallel to the direction of growth (vertical) in the flattened front than in the normal simulation. A natural question is how does cellular alignment affect *P*_surf_ independently of the roughness? To answer this question, we simulated a modified model, in which external torque *τ* = − *τ*_max_sin[(*ϕ* − *ϕ*_preferred_) mod *π*] was applied to the cells, forcing them to align preferentially in the direction *ϕ*_preferred_. We investigated two forced alignments: *ϕ*_preferred_ = 0 corresponding to cells parallel to the *x*-axis and hence to the growing edge of the colony, and *ϕ*_preferred_ = *π*/2, which corresponds to the vertical orientation of cells (perpendicular to the growing edge).

Figure 10*b* compares these two different modes with previous simulations with no external torque, for approximately the same thickness and roughness of the growing layer. It is evident that the orientation of cells strongly affects the surfing probability: horizontally forced cells have around 3 × smaller *P*_surf_ compared with the normal case, which in turn has *P*_surf_ ∼ 5× smaller than vertically forced cells.

#### 4.2.5. Shorter cells have higher *P*_surf_ than long cells

To check how the aspect ratio of cells affects *P*_surf_, we simulated cells whose maximal length was only 2 μm and the minimal separation before the spherical caps was zero, i.e. the cells became circles immediately after division. As before, we selected a set of *k*'s such that the thickness and roughness were approximately the same for all simulations. In order to make a fair comparison between ‘short rods’ and ‘long rods’ from previous simulations, thickness and roughness were expressed in cell lengths rather than in micrometres. This was done by dividing both *h* and *ρ* by the average length of a cell measured for cells from the growing layer. Figure 10*c* shows that short rods have a much higher surfing probability than long rods.

In all previous simulations, even for short rods, cells remembered their orientation from before division and growth always initially occurred in that direction. To see whether this has any impact on *P*_surf_, we considered a scenario in which the new direction of growth is selected randomly and does not correlate with the direction prior to division. Figure 10*c* shows that *P*_surf_ almost does not change regardless whether a short cell randomly changes its orientation after division or not.

### 4.3. Surfing probability and the mechanical properties of bacteria

Our results from the previous section demonstrate that surfing is affected by (i) the roughness of the growing layer, (ii) the orientation of cells, and (iii) the thickness of the growing layer if mutations occur inside the growing layer and not only at its edge. To show this, we varied thickness, roughness and orientation of cells by using ad hoc external forces flattening out the front or forcing the cells to order in a particular way. In this section, we investigate what parameters of the model affect surfing in the absence of such artificial force fields.

#### 4.3.1. Thickness of the growing layer

If cells are prohibited to form multiple layers, as in our two-dimensional simulations, thickness *h* can be determined from the parameters of the model by a simple dimensional analysis. Assuming that *h* is proportional to the characteristic scale over which the nutrient concentration and cell density reaches bulk values [17], we can approximate *h* by4.4
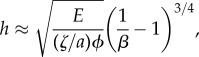
where *E* is the elastic modulus of the bacterium (Pa), *a* is the average area per cell (μm^2^), *ζ* is the friction coefficient (Pa h), *ϕ* is the replication rate (h^−1^) and *β* < 1 is a dimensionless ratio of the nutrient consumption rate to the biomass production rate (i.e. new bacteria): *β* = (*kρ*_0_)/(*ϕc*_0_). Equation (4.4) shows that thickness *h* increases with increasing cell stiffness (larger *E*) and replication rate *ϕ*, and decreases with increasing nutrient uptake *k* and increasing friction *ζ*. The aspect ratio of the cells does not affect *h* in our model. Equation (4.4) suggests that the thickness of the growing layer can be conveniently controlled in an experiment by varying temperature or growth medium (both of which affect the growth rate), or by varying the nutrient concentration *c*_0_. We shall use the first two methods when discussing the experimental verification of our theory.

#### 4.3.2. Orientation of cells

A useful measure of the global alignment of cells in the colony is the order parameter *S* = 〈cos^2^(*ϕ* − *Φ*)〉. Here, *ϕ* is the angle a cell makes with the *x*-axis and *Φ* is the angular coordinate of the vector normal to the front; this is to remove a trivial contribution to *S* due to the curvature of the front caused by roughness. According to this definition, *S* = 1 if all cells are perfectly vertically aligned (in the direction of growth), *S* = 0 if they are horizontal (parallel to the front) and *S* = 1/2 if their orientations are random. It turns out that changing the uptake rate (and hence thickness *h*) from *k* = 1.6 to *k* = 2.8 changes *S* by a small amount from *S* = 0.77 to *S* = 0.70. Here, we are more interested in other factors that do not affect *h*.

#### 4.3.3. Friction

One such factor is the nature of friction between cells and the substrate. So far, in all simulations the friction force was proportional to the cell's velocity, irrespective of the direction of motion. To test whether this assumption affected front roughness and the surfing probability, we ran simulations in which friction coefficients were different in the directions parallel and perpendicular to the cell's axis. We replaced equation (2.1) for the dynamics of the centre of mass with the following equation:4.5
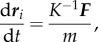
where the matrix *K* accounts for the anisotropy of friction:4.6
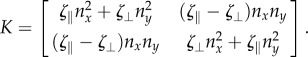
We now have two friction coefficients: *ζ*_⊥_ is the coefficient in the direction perpendicular to the cell's major axis ***n***, whereas *ζ*_∥_ is the coefficient in the parallel direction. For convenience, we shall assume that *ζ*_∥_ = *Aζ*, *ζ*_⊥_ = *ζ*/*A* where *A* is the ‘asymmetry coefficient’ and *ζ* is the isotropic friction coefficient, same as in previous simulations (table 1). For isotropic friction, *A* = 1; hence *ζ*_⊥_ = *ζ*_∥_≡*ζ* and *K* = **1***ζ*, and we recover equation (2.1). If *A* > 1, it is easier for the rod to ‘roll’ than to slide along the major axis. If *A* < 1, it is easier for the rod to slide.

**Figure 11.**
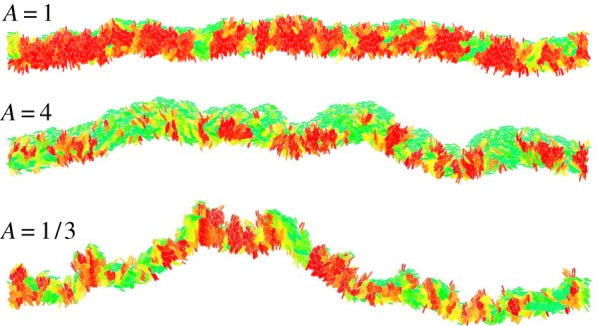
Snapshots of a growing colony with different friction anisotropy *A*. The global order parameter *S* = 0.79 (isotropic friction *A* = 1), *S* = 0.53 (rolling rods *A* = 4) and *S* = 0.63 (sliding rods *A* = 1/3). See figure 10 for the key. (Online version in colour.)

**Figure 12.**
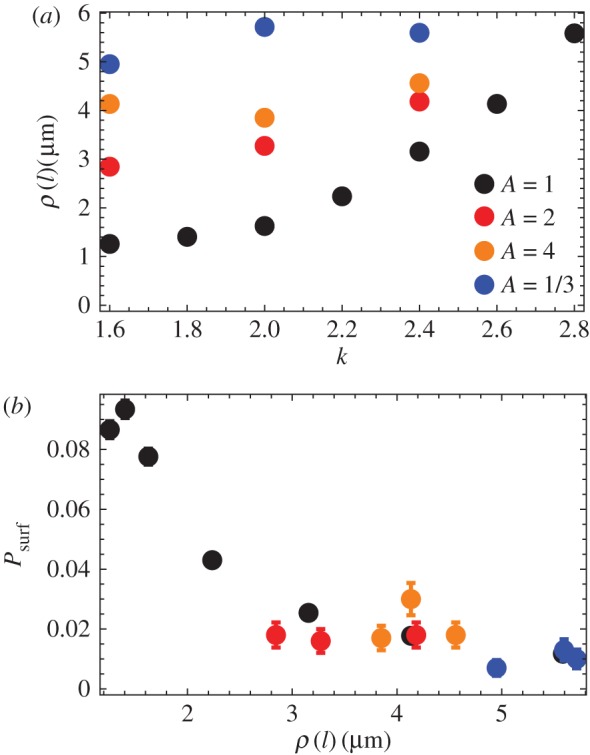
(*a*) Local roughness *ρ*(*l*) as the function of *k*, for different levels of friction anisotropy: no anisotropy (black points, *A* = 1), ‘rolling rods’ *A* = 2 (red), *A* = 4 (orange) and ‘sliding rods’ *A* = 1/3 (blue). (*b*) Surfing probability versus local roughness *ρ*(*l*) for the same parameters as in panel (*a*). In all cases, *L* = 320 and *l* = 80 μm. (Online version in colour.)

Figure 11 shows images of the front for different levels of friction anisotropy. In the anisotropic ‘rolling rods’ case (*A* > 1), cells are significantly more oriented edge-on to the colony, and the roughness is noticeably larger. In the ‘sliding rods’ case (*A* < 1), the roughness is even larger but the orientation of cells falls between the isotropic and the ‘rolling rods’ case. This is quantified in figure 12*a*, where we plotted the local roughness *ρ*(*l*) as a function of *k*, for a fixed *l* = 80 μm. Figure 12*b* shows that, as expected, the surfing probability goes down with increasing local roughness.

## 5. Comparison with experiments

**Figure 13.**
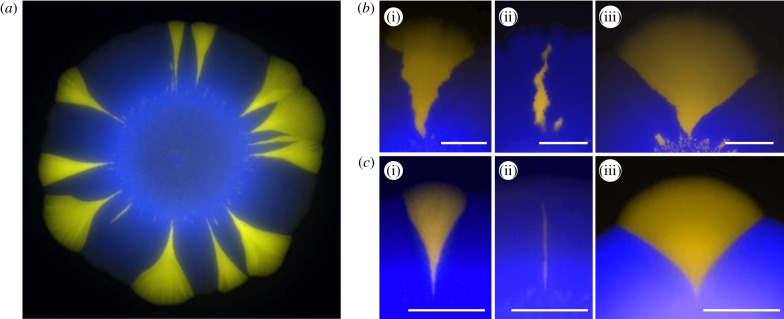
(*a*) An example of an *S. cerevisiae* colony with beneficial mutants (yellow) forming sectors. The mutants have a growth rate advantage of *s* ≈ 10%. (*b*,*c*) Fate of mutant cells—experimental counterpart of figure 5. Colonies of *E. coli* (*b*) and *S. cerevisiae* (*c*) were inoculated using a mixture of a majority of wild-type cells (blue, false colour) and a small number of mutant cells (yellow) with *s* = 8% (i and ii). Some mutant clones formed large sectors (i), while others (ii) lagged behind the front, became engulfed by wild-type cells and eventually ceased to grow (bubbles). A large growth advantage (*s* ≈ 16%, iii) caused the sector to ‘bulge out’. All three phenomena are well reproduced by our simulations (c.f. figure 5). In all panels, scale bar, 2 mm. (Online version in colour.)

We next checked whether the predicted dependence of the surfing probability on the roughness of the growing layer agree with experiments. We measured surfing probabilities of beneficial mutants with different selective advantages *s* = − 5 … 25% in colonies of *E. coli* and *S. cerevisiae* (Methods) grown at different conditions affecting the roughness of the growing layer. A small number of fluorescently labelled mutant cells was mixed with a much larger number of wild-type cells, and a small droplet of the mixture was used to inoculate a colony on a Petri dish. After a few days, colonies with a characteristic sectoring pattern emerged (figure 13). By zooming into the colony edge, we confirmed that some mutants ‘surfed’ at the front and expanded into large sectors, whereas some mutants did not make it and became trapped as bubbles in the bulk of the colony (figure 13; cf. figure 5).

**Figure 14.**
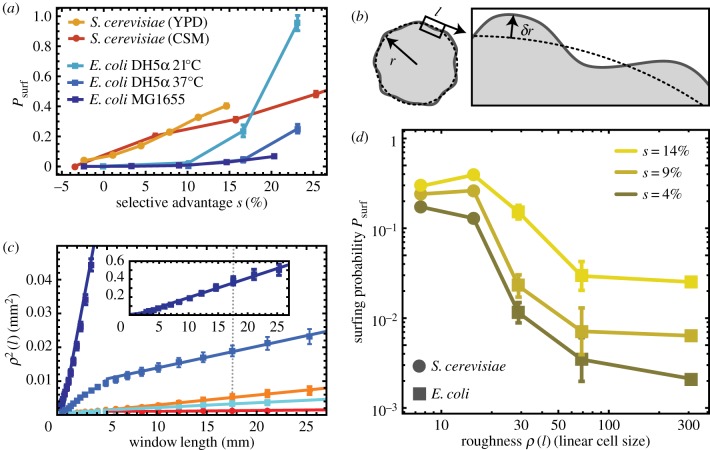
Surfing probability versus roughness in experimental colonies. In all panels, squares and circles correspond to *E. coli* and *S. cerevisiae*, respectively. (*a*) Surfing probability *P*_surf_ for different species and growth conditions as a function of the selective advantage *s*. *S. cerevisiae* has a much higher *P*_surf_ at low *s*, while *P*_surf_ of *E. coli* strain DH5*α* at 21°C increases faster than linearly for large *s*, surpassing *S. cerevisiae* for *s* > 15%. (*b*) Diagram illustrating how roughness *ρ*(*l*) was measured (Methods). (*c*) The local roughness squared *ρ*^2^(*l*) for different conditions (colours as in (*a*); error bars are standard errors of the mean over at least 10 colonies per condition). Solid lines are linear fits to the data points. The dotted line corresponds to the window length *l* = 17 mm used to calculate roughness in panel (*d*). The inset shows *ρ*^2^(*l*) for *E. coli* MG1655 (dark blue), which has the highest roughness. (*d*) Surfing probability versus *ρ*(*l* = 17 mm, for different *s*. To compare *E. coli* and *S. cerevisiae*, we normalized roughness by the linear cell size (square root of the average area), which we estimated from microscopic images to be 2 and 4.7 μm, respectively. (Online version in colour.)

We counted the number *N*_sec_ of sectors and estimated the surfing probability *P*_surf_ from the following formula [25]:5.1
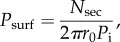
where *P*_i_ is the initial fraction of mutant cells in the population and *r*_0_ is the initial radius of the colony (in units of cell diameters). Note this equation makes sense only if surfing is restricted to the first layer of cells; we have shown that this is true in computer simulations and we shall experimentally validate it later in this section. Figure 14*a* shows *P*_surf_ for *E. coli* and *S. cerevisiae*, and for different conditions. In the limit of low selective advantage *s* < 10% which we focus on here, the surfing probability is highest in colonies of roughly spherical *S. cerevisiae*, which have rather smooth boundaries, and smallest for the rod-shaped bacterium *E. coli*, characterized by rough fronts. This agrees with our predictions (figure 10); however, it does not yet show whether this is due to difference in the cell shape (aspect ratio; cf. the penultimate paragraph of §4.2) or different thickness or roughness of the growing layer.

To study the connection between surfing and surface roughness, we computed the local roughness *ρ*(*l*) as a function of window length *l* (figure 14*b*; cf. equation (4.3) and Methods) for the same colonies for which we previously calculated *P*_surf_ (figure 14*a*). In all cases, *ρ*^2^(*l*) showed a linear dependence on window length *l* after a transient at small window lengths, i.e. the colony boundary behaved like a standard random walk (figure 14*c*).

We then tested the correlation of colony roughness with surfing probability in a similar way to what we did in computer simulations. In figure 14*d*, we plot the surfing probability *P*_surf_ as a function of colony roughness measured at one specific window length *l* = 17 mm (dotted line in figure 14*c*), for different selective advantages *s*. We observe that the surfing probability of *E. coli* decreases with increasing roughness (figure 14*d*) for all *s*, in good qualitative agreement with our simulations. Similar results are obtained for different choices of the window length *l* for which roughness is calculated. The situation is less clear for *S. cerevisiae*; we hypothesize that this is due to roughness being too small (cf. figure 9) to markedly affect the surfing probability.

**Figure 15.**
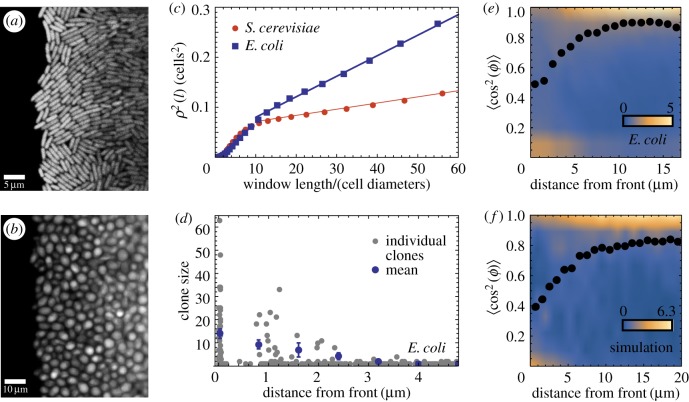
Microscopic properties of the growing layer. (*a*,*b*) Snapshot of an *E. coli* front (*a*, scale bar 5 μm) and an *S. cerevisiae* front (*b*, scale bar 10 μm) front. (*c*) Local roughness squared *ρ*^2^(*l*) as a function of the window size *l*. Dashed lines are fits to the data points. (*d*) The number of offspring for all initial cells near the front, for *E. coli*. Only cells within 2–3 μm (approx. one cell) from the edge of the colony have a significant number of offspring. (*e*) Probability density *P*(*S*) of the order parameter *S* = 〈cos^2^*ϕ*〉 for *E. coli* as a function of the distance from the edge. Blue, low probability; yellow, high probability (cf. the scale bar). *P*(*S*) has been normalized so that
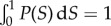
. The dotted line is the average order parameter versus the distance from the front. Cells are preferentially aligned with the direction of propagation, except for cells directly at the front, which are parallel to it. (*f*) Density plot of the order parameter for a simulated front with *k* = 1.4, *L* = 320 μm. (Online version in colour.)

We next examined how microscopic properties of the front (cellular orientation) correlated with macroscopic roughness. We analysed microscopic images of the fronts of *E. coli* and *S. cerevisiae* fronts (Methods, data from [25]), and measured local roughness *ρ*(*l*) over submillimetre length scales *l*. Example snapshots in figure 15*a*,*b* show that roughness of the fronts indeed differ very much for these two microorganisms. Figure 15*c* confirms that *E. coli* has a much higher roughness compared to *S. cerevisiae*, suggesting that macroscopic roughness on the colony scale is a consequence of microscopic front roughness on the single-cell level.

To study the dynamics of surfing, we tracked *E. coli* cells over 200 min and measured their distance from, and orientation relative to the edge of the colony, as well as the number of offspring for all cells in the initial image. Figure 15*d* shows that cells only have an appreciable number of offspring if they are within about one cell diameter of the front. This agrees with our conclusion from simulations and justifies inserting mutants only directly at the front.

Figure 15*e* shows the order parameter *S* = 〈cos^2^(*ϕ* − *Φ*)〉, which measures the orientation of cells and has been defined in §4.3, as a function of the distance from the front. Cells near the front tend to align parallel to the front. This changes quickly behind the front, with most cells being perpendicular to the growth direction starting about 5 μm behind the front. Figure 15*f* shows the distribution of *S* obtained from simulations; the agreement with the experimental data from figure 15*e* is excellent, suggesting that our model indeed captures the dynamics of the growing bacterial front reasonably well.

## 6. Conclusion

In this work, we have focused on the role of mechanical interactions in microbial colonies. We first used computer simulations to show that the speed of biological evolution, measured by the probability that a new mutation ‘surfs’ at the growing edge of a microbial colony, depends mostly on the thickness and roughness of the growing layer of cells at the colony's front. Thicker fronts decrease the per-cell surfing probability because only cells from the very first layer of cells create successful progenies, and the fraction of such cells decreases with increasing front thickness. Rougher fronts also decrease the surfing probability for a similar reason; only cells at the tips of the front's protrusions are successful and these tips become smaller for rougher fronts. Moreover, roughness and thickness are related; thicker front have lower roughness and vice versa. While the dependence between genetic segregation and the front thickness [47], and between thickness and roughness [48] has been known previously, in this work we have shown that it is actually the roughness of the growing layer that should be thought of as affecting the surfing probability in the causal sense. We have also linked thickness and roughness to the mechanical properties of cells for the first time. Moreover, we have discovered that the orientation of cells has also a significant effect, irrespective of front roughness, on the surfing probability. Finally, we have confirmed some of our predictions (surfing probability versus front roughness and the orientation of cells versus distance from the front) in experiments in which we varied the growth rate and the type of cells.

All three quantities, front thickness, front roughness and cellular alignment depend in a very non-trivial way on the properties of cells and their environment: cell-surface friction (and anisotropy of thereof), elasticity of cells, their growth/nutrient uptake rate and their shape. Many of these parameters are very difficult to control experimentally without affecting other parameters. To properly disentangle the effect of the shape of cells, friction, growth rate, etc., on the surfing probability, further experiments are required in which these factors are varied independently. For example, the shape of *E. coli* can be varied by using MreB mutants [49]; while this often also affects the growth rate [50], an experiment with round *E. coli* MreB mutants could complement our results in an interesting way.

Microbial evolution is a research area that is important both from fundamental and practical viewpoints. In particular, our research shows that mechanical forces such as friction can play a significant role in biological evolution of microorganisms. To our knowledge, this article is the first that not only puts forward this idea but also provides concrete arguments in its support.

From a more practical point of view, our results are relevant to the evolution of antimicrobial resistance. It has been demonstrated that even a small bacterial population can develop *de novo* resistance to some antimicrobial drugs in less than a day [51]. This rapid evolution makes the most popular drugs—antibiotics—increasingly ineffective [52]. Since the rate of discovery of new antibiotics has steadily declined over years [53], the evolution of drug-resistant bacteria has been highlighted as one of the major challenges we will face in the coming decades. By demonstrating the role of mechanical interactions on biological evolution in microbial aggregates, our research opens up a new antimicrobial paradigm in which the physical properties of microbes could be targeted alongside standard antimicrobial therapy to reduce the probability of evolving resistance to drugs.

## Endnote

1 Alternatively, *h* can be defined as the area of the colony that contains replicating cells divided by the interface length *L*. Both methods produce similar results.

## Data accessibility

All simulation/experimental datasets and code are available from Edinburgh DataShare (http://dx.doi.org/10.7488/ds/1702).
